# The radiographic method for evaluation of axial vertebral rotation – presentation of the new method

**DOI:** 10.1186/1748-7161-9-11

**Published:** 2014-08-01

**Authors:** Pavel Cerny, Ivo Marik, Iveta Pallova

**Affiliations:** 1 Faculty of Physical Education and Sport, Charles University, Prague, Jose Martiho 31 16252 Prague 6, Czech Republic; 2 Ambulant Centre for Defects of Locomotor Apparatus l.l.c., Olsanska 7, 13000 Prague 3, Czech Republic; 3 ORTOTIKA l.l.c., Area of University Hospital in Motol, V Uvalu 84, 15006 Prague 5, Czech Republic; 4 Faculty of Medical Studies, West Bohemia University, Pilsner Univerzitni 2732/8, 306 14, Pilsner, Prague, Czech Republic

**Keywords:** Axial vertebral rotation, Radiographic method, X-ray of spine, Vertebral rotation device

## Abstract

The objective of this study is to present a new radiographic method for the assessment of vertebral rotation from an antero-posterior view of conventional X-rays which is sufficiently precise in comparison with radiographic methods presently used in clinical practice (methods of Nash-Moe and Perdriolle). This method is based on the properties of the geometric shape of vertebrae and their shared dimensional proportions. It means that the relation between vertebral body width and height doesn’t change significantly within the entire thoracic and lumbar sections of the spine. In order to verify the method, we have constructed a special device for vertebral fixation. Subsequently, the X-ray pictures of individual human vertebrae with predefined rotation values (ranging from 0 degrees to 45 degrees by steps of 3 degrees) were radio-graphically measured and then compared with their actual axial rotation on the vertebral rotation device. All arithmetic averages correlate very closely with the actual values. The verification of axial vertebral rotation with the assistance of CT and MRI pictures of six scoliotic patients (in supine position) and the evaluation of axial vertebral rotation by both the new radiographic method and with the Perdriolle method proved the satisfactory accuracy of our method. The main advantage of the newly presented radiographic method is the uncomplicated measurement of vertebral rotation from AP projection of conventional X-ray pictures or from its printed copies. The gold standard of the new radiographic method is the evaluation of axial rotation of vertebrae to 30 degrees approximately and the shape of vertebral bodies without severe structural deformities. The new radiographic method seems to be suitable for use in clinical practice.

## Introduction

There is a great need for knowledge about vertebral rotation in patients suffering from scoliosis in both preoperative and postoperative assessment, providing better appreciation of the impact of bracing or surgical interventions. Scoliosis is an idiopathic spine deformity which during growth causes adaptive changes of vertebrae in the main anatomic planes and is defined very rigorously with unified terminology [1]. The assessment of rotation of a spinal segment on the transverse plane is difficult. Accurate values of vertebral rotation are also an important requirement for the development of biomechanical models of the spine.

The severity of scoliosis is routinely evaluated by conventional X-ray pictures in antero-posterior (AP) and lateral projection in situ. The Cobb angle of spine curvatures is the most widely used method in our country. It is very easy, objective and adequately accurate [2-5]. There were many methods proposed and used to measure vertebral rotation such as radiography [6,2-12], CT [13-16], MRI methods [17] and ultrasound [18]. Radiographic methods such as Nash’s and Moe’s [8], Perdriolle’s [9] and Raimondi’s [12] are still used by clinicians for measurement of the thoracic and lumbar vertebrae rotation in conventional X-rays. These methods are not sufficiently precise in comparison with measurements obtained in CT or MRI scans.

In AP projection of conventional X rays [2,6,3-11] it is possible to evaluate the axial rotation of vertebra indirectly. The rotation of vertebra is visible on X-ray film (AP) by a change of the positional details of a vertebral body. For example Cobb’s method for vertebral rotation uses the position of spinous processes [2]. The disadvantage of the spinous process methods are that spinous processes are difficult to visualize on real X-ray films [8]. The methods of Nash-Moe, Perdriolle and Raimondi [8,9,12] use the position of pedicles. The methods of Nash-Moe [8] and Perdriolle [3,9,12] are still the most used radiographic methods in clinical practice.

The Nash-Moe method is very approximate. It recognizes only 5 levels (0 – 4). To evaluate the level of vertebral rotation more accurately it is better to use the methods of Perdriolle (Perdriolle torsion-meter) or Raimondi (Raimondi pattern). Perdriolle torsion-meter shows vertebral rotation in 5° increments. The Raimondi pattern is made in 2° increments. Both methods give the actual level of vertebral rotation in degrees, the accuracy of these methods was compared by Weiss [12]. Both of these methods need life-size X-ray pictures.

Computer tomography is becoming popular for assessing axial rotation. Aaro and Dahlborn [13] and Ho et al. [15] developed techniques of rotation measurement from CT images. But the CT examination presents a considerable radiation loading. Recently some authors presented computer methods [11,19] to measure vertebral rotation from AP projection. The methods need a lot of parameters to be set i.e. spine and vertebral proportions in order to be functional and precise and to find relevant principles for computers programs. Nevertheless the methods need digital X-ray pictures and accurate software to evaluate the angle of vertebral rotation.

Despite many advantages of above mentioned technologies, radiographic methods remain the most cheap, safe and common and are used for accurate comparison of newly developed techniques (10). Radiographic measures are obtained routinely in a standing posture whereas CT measures are obtained in a supine position. It is noteworthy that scoliotic curves appear less severe in the supine position, both in terms of curvature and rotation.

The authors have more than 20 years’ experience with the comprehensive treatment of adolescent idiopathic scoliosis especially with dynamic bracing and its new models, they achieved meaningful therapeutic results. Therefore the development of a precise radiographic method for the evaluation of axial vertebral rotation was influenced by their daily usage. The main advantage of the newly presented radiographic method is the uncomplicated measurement of vertebral rotation from AP projection of conventional X-ray pictures or from its printed copies. Simple common tools (pencil, ruler, protractor) are only used. The vertebral rotation is directly measured in degrees. The absolute size of an X-ray picture or its copy is not important.

The authors present their own new radiographic method that is sufficiently precise for the measurement of vertebral rotation from conventional X-rays and is easily practicable in clinical practice.

## Material and methods

**Figure 1:**
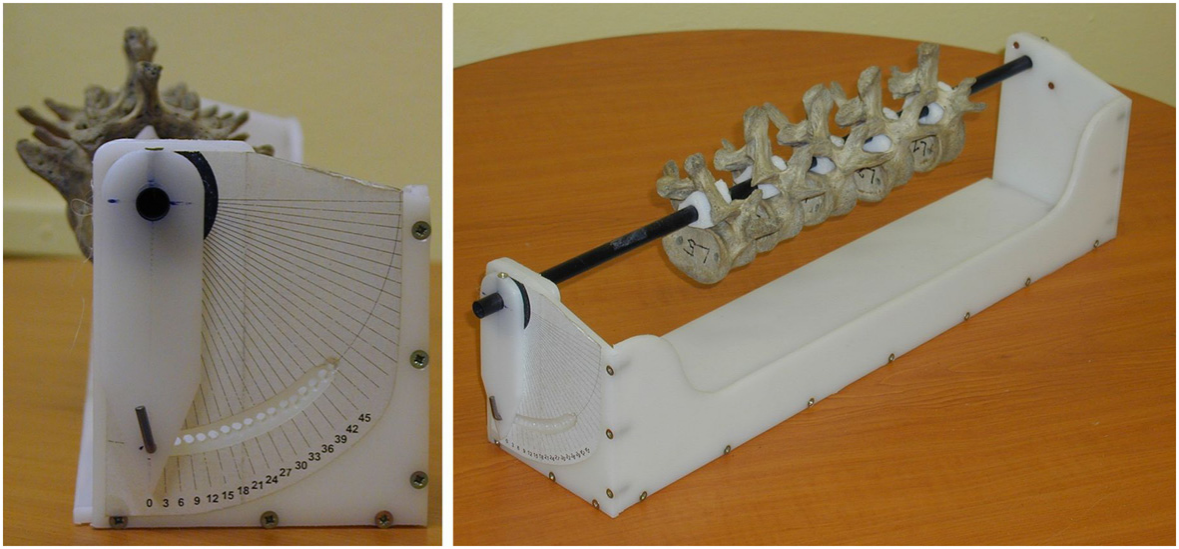
Vertebral rotation device (made by author), 3 degree rotation steps.

The main aim of our work was to determine axial rotation of the vertebra from AP projection with the assistance of graphical principles. The new radiographic method of quantifying axial vertebral rotation is based on geometric shape properties of the vertebrae and their mutual dimensional proportions ([20] (table 1–5, p. 29)). It is necessary to mention that the biomechanical terminology [20] is different to the terminology of the Scoliosis Research Society (SRS) according to Stokes [1]. Both terminologies have different orientation of axis. Henceforth the SRS’s orientation will be used [1].Primarily a vertebral rotation device was made to compare particular readings of X-ray film with real known pre-defined values. The vertebral rotation device allows set rotation from zero to 45 degrees in 3 degree rotation increments, Figure 1.

**Figure 2:**
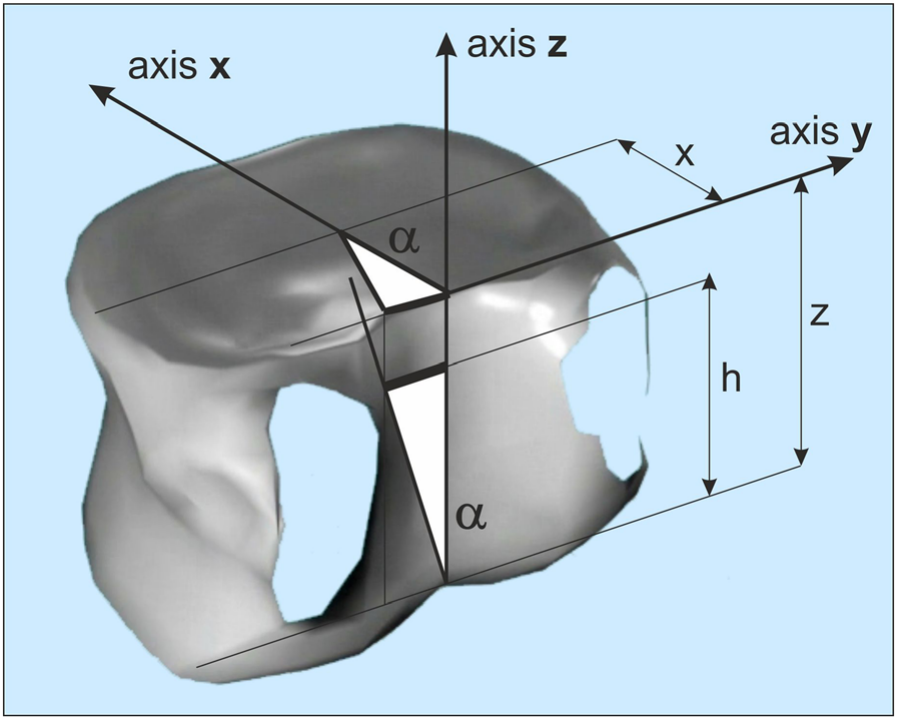
**The basic idea of radiographic method.** Radius of rotation x is in a correlation with the height of vertebral body z.

The method was verified on 5 non-deformed human lumbar vertebrae of the same spine column and subsequently on non-deformed human thoracic and lumbar vertebrae of other individuals. The vertebrae were provided courtesy of both the Faculty of Science and the Faculty of Physical Education and Sport, Charles University in Prague.The method is based on the presumption that the diameter of measurable geometric rotation in the transversal plane is in a correlation with the height parameter of the vertebra. The basic idea of this method is illustrated on Figure 2.

The process of first axial vertebral rotation evaluation: The locations of pedicle shadows (inner or outer contour or middle of pedicles) were chosen as in the other previously mentioned methods [7,8,10,11]. It would be advantageous to take the inner contours of pedicles. Two X-ray pictures of thoracic vertebra Th6 with rotation 21° and the lumbar one of vertebra L2 with rotation 21° were tested during the first stage. Perpendiculars were placed through the middle of the vertebral body and through the pedicle’s middle to the base of the vertebral body as shown in both pictures as the second step. The apex of the angle of 21° was placed towards the centre of the vertebral base so that one leg of the angle was adjacent to the perpendicular through the centre of the vertebral body and the second one crossed the perpendicular of the pedicle’s centre. The crossover was at almost the midpoint of vertebral body height of lumbar vertebra and close to ¾ of vertebral body height of thoracic vertebra. These principles were tested on all X-ray pictures with rotation from 0 to 45 degrees. The comparison was closely relevant. The idea was shown as applicable and following this it had to be verified, compared and demonstrated.

The variety of average dimensions of thoracic vertebrae [20], which are visible during AP projection of conventional films show that the average aspect ratio of vertebral widths to heights is less than 9%. The aspect ratio of radiuses (x = LED/2 + SCD/2); [20]) in the transversal plane to vertebral heights is less than 7%. It is possible to use the same algorithm for all thoracic vertebrae. There is a similar situation for lumbar vertebrae L1 to L4. They are very similar to each other. It seemed that vertebra L5, which is a little lower to the other lumbar vertebrae, would need its own algorithm too, but the experiment showed that thoracic succession could be likewise [6] applied to vertebra L5.

The non-rotated centre of the distance between the pedicles lies on the same sagittal plane as the centre of vertebral body width. If the vertebra is rotated the pedicle’s centre and vertebral body centre will recede. The distance between both centre points is named d. It represents the axial vertebral rotation on radius x. It is generally valid that d = x* tan α, for small angles tan α ~ α [m, rad] by goniometric functions (differences: 0.10° on angle 10°, 0.35° on angle 15°, 0.85° on angle 20°, 1.72° on angle 25° and 3.08° on angle 30°). It follows the accuracy of the radiographic method. It deteriorates with degree of rotation. The magnitude of error is very favourable up to 20° and acceptable to 30° for clinical practice.

**Figure 3:**
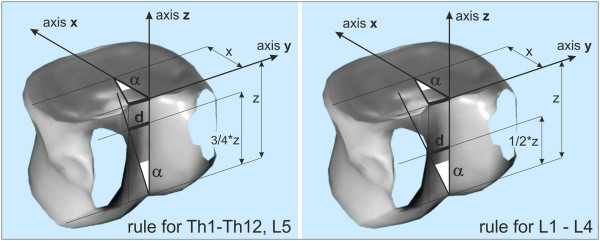
**Principles for rules of radiographic evaluation of axial vertebral rotation.** There are two bit different rules for thoracic spine and vertebra L5 (x = 0.75*z) and for lumbar spine L1 to L4 (x = 0.5*z).

**Figure 4:**
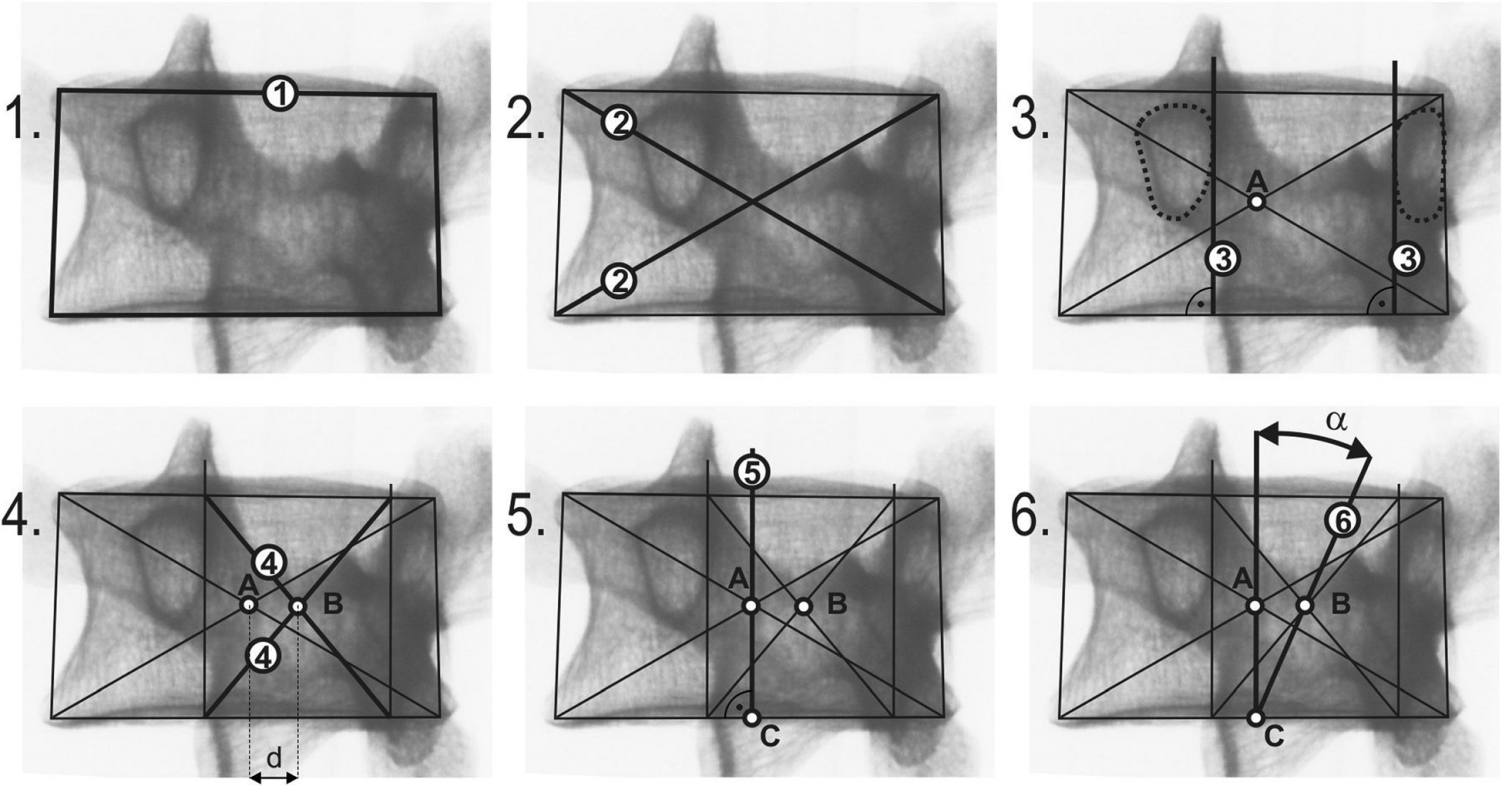
Phased geometrical procedure to evaluate axial vertebral rotation angle α of lumbar vertebra L1 to L4, steps 1 to 6.

**Figure 5:**
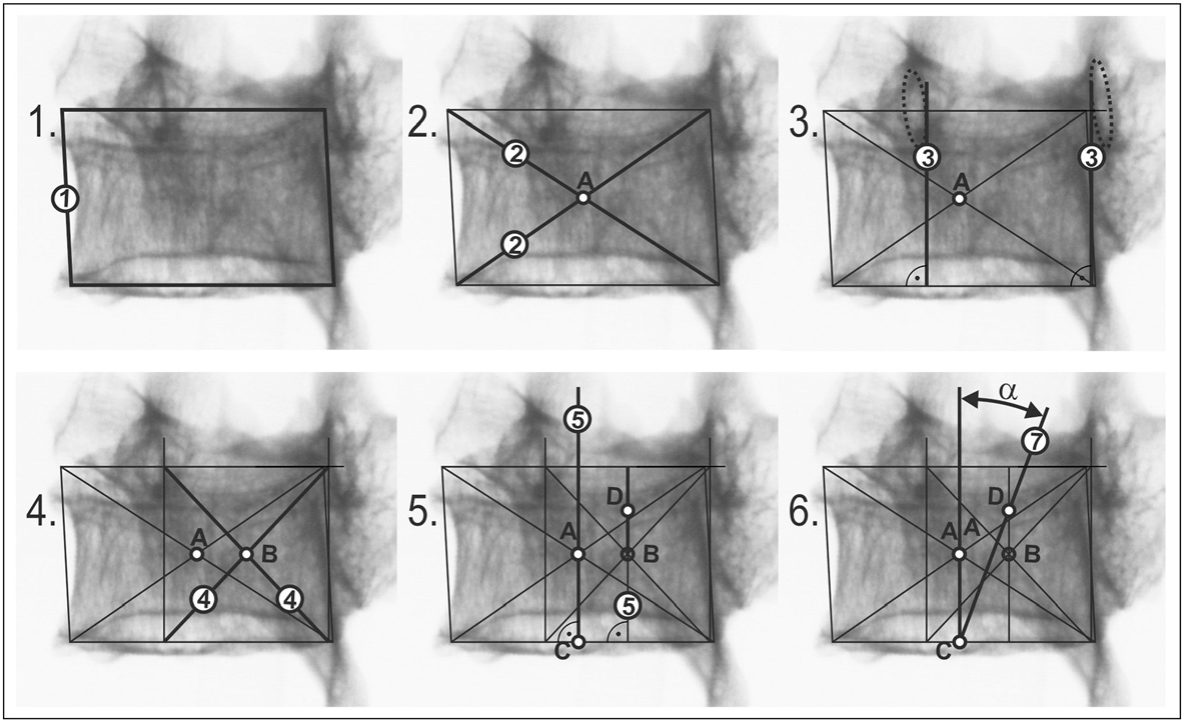
Phased geometrical procedure to evaluate axial vertebral rotation angle α of thoracic vertebrae and lumbar vertebra L5, steps 1 to 6.

Thoracic and lumbar vertebrae have different aspect ratio of radius x to height z (x/z_(Th)_ = 1.21 and x/z_*L*1−4_ = 0.86; x = LED/2 + SCD/2; z = VBHp [20]). The verification of all tested vertebrae in all steps of rotation confirmed the criterions for thoracic spine and vertebra L5 x = 0.75*z [m] and for lumbar spine x = 0.5*z [m], Figure 3.The procedure which is applicable to lumbar vertebrae is illustrated in Figure 4. The procedure applicable to thoracic vertebrae is illustrated in Figure 5. The basic point was selected as the centre of the width of the projection of a vertebral body A. It is simply obtained by copying the outline 1 of the vertebral body or by drawing compromise rectangles or rhomboids over pictures of deformed vertebrae and marking their diagonals 2. The centre of the distance between the pedicles is determined by drawing perpendiculars 3 from the bottom edge of the vertebra at the point of the interior projection of pedicles (all perpendiculars are given as perpendiculars to the bottom edge of the vertebra). The points of the intersection of the perpendiculars and the straight line from the top and bottom edge of the vertebral projection constitute another quadrangle very similar to a rectangle. By drawing diagonals 4 of this rectangle, we obtain the required centre of the distance between pedicles B. Thus, we have determined the distance d. Another perpendicular 5 is drawn from the bottom edge of the vertebra through the centre of the projection of the vertebral body A. The point of intersection of this perpendicular with the bottom edge of the vertebra is shown as C. From this point, due to the proportions of the lumbar and thoracic vertebrae, we must adopt a different procedure for the thoracic and lumbar sections of the spine.In the case of lumbar vertebrae, the axial vertebral rotation angle is the angle between the perpendicular 5 leading through the centre of the vertebra A and the straight line 6 leading through points B and C. The whole procedure is described in stages in Figure 4.

In the case of thoracic vertebrae, it is necessary to find point D, obtained on the perpendicular from the bottom edge of the vertebra, leading through point B, by dividing in half the distance between point B and the top edge of the vertebra. This can be measured or just estimated without the risk of any significant error for determining the axial rotation angle.The identified point D corresponds to ¾ of the vertebral body height. The axial rotation of thoracic vertebrae and L5 vertebra is the angle between the straight line leading through point A – straight line 5 and the straight line leading through points C and D – straight line 7. The particular steps of the procedure are described in Figure 5. There is an important rule for observing the invisible pedicle shadow which is merged with the vertebral body contour in larger axial rotation - usually more than 30°. There is an important rule for observing the invisible pedicle shadow which is merged with the vertebral body contour in larger axial rotation. When the shadow of pedicle is not visible in the concave site, the contour of the vertebral body on the concave site instead of the pedicle contour will be visible. We suppose that the pedicle coincides with the vertebral body contour and the larger axial rotation - usually more than 30° - does not change its location.

**Figure 6:**
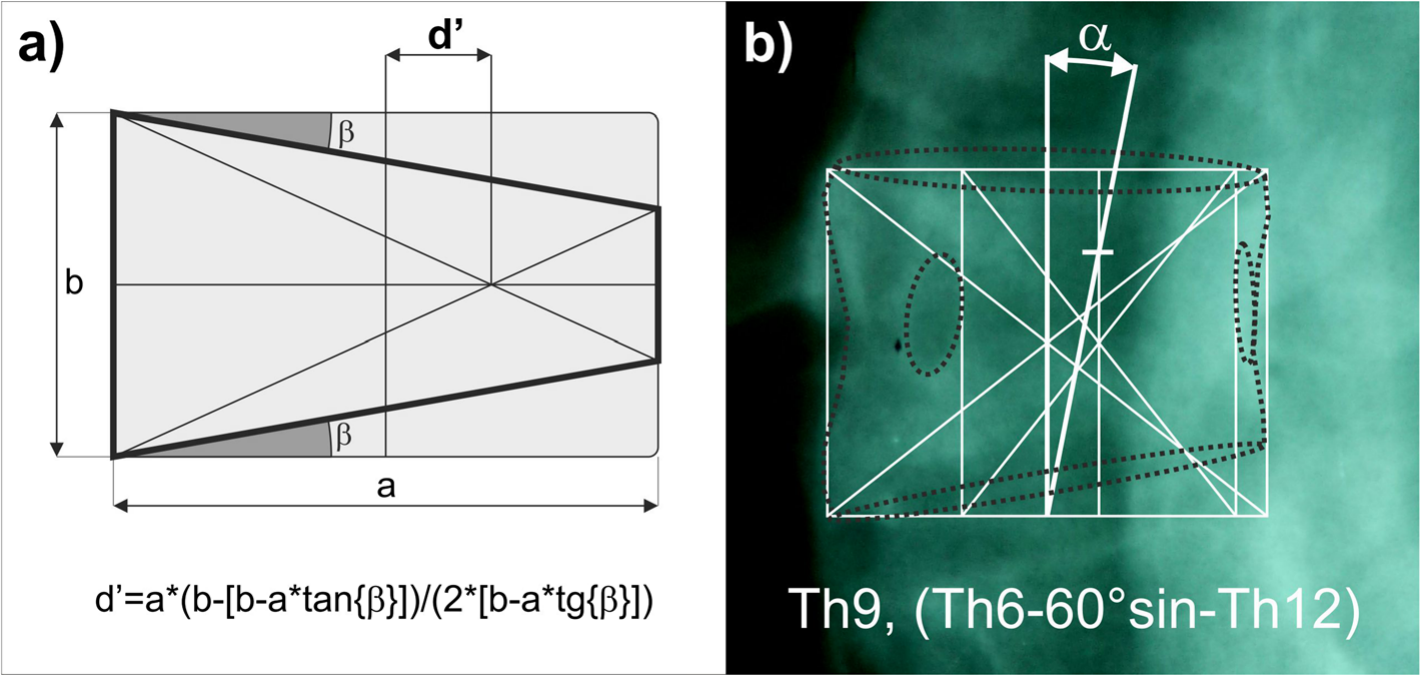
**Wedged vertebra.** The drift d’ of vertebral body centre of wedged vertebra according to standard geometrical principles **(a)**. Supplying procedure on wedged vertebra where it is supposable that the vertebral body basis is parallel to pedicle’s suture **(b)**.

**Figure 7:**
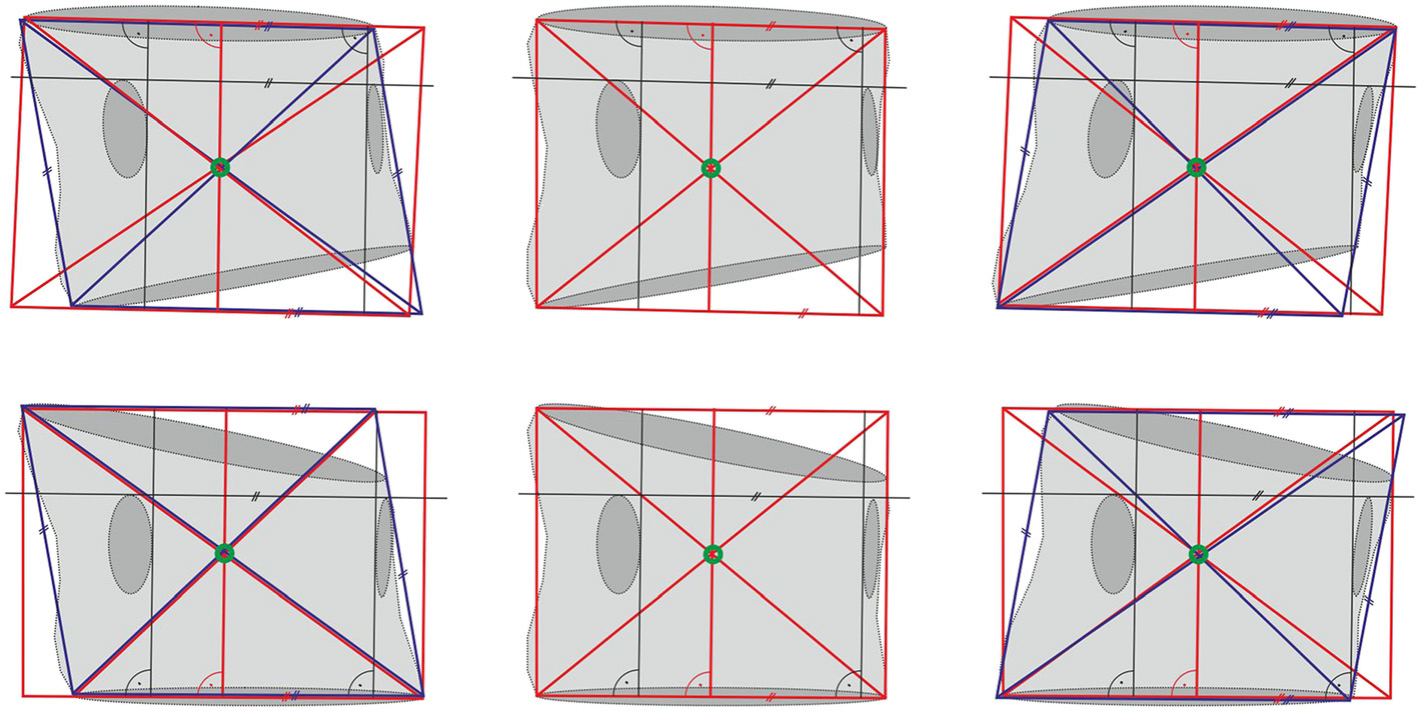
Examples of drawing compromised rectangles (red colour) and rhomboids (blue colour).

Local axial vertebral rotation is different to global spine axis [1]. The declination of vertebral body on a sagittal plane is displayed as an ovoid shadow on the frontal plane, but the width and height of the vertebra’s body is visible. The width of the vertebral body is decreased by the declination of the vertebra only slightly without observable influence to aspect ratio of vertebral width to heights.The vertebrae of severely affected scoliotic spines usually have local deformity and asymmetry. Severely affected structural scoliotic curves of the thoracic spine are shown as wedged vertebral bodies on X-ray pictures. The graphical centre of a wedged vertebral body drifts to the apex of the wedge according to standard geometrical principles, Figure 6a, and right substitute procedure on wedged vertebra, Figure 6b. It is supposable that the vertebral body basis is parallel to pedicle’s suture.As it is necessary to find both the centre of the vertebral body width on its base and the centre of the pedicle’s position, it is necessary to draw compromise rectangles or rhomboids relating to convex height over pictures of wedged vertebrae, Figure 6b. Preservation of habitual ratio height/width. The convex site isn’t deformed by compression. The base should be parallel to the pedicle’s suture. We presume that a small deviation in parallelism isn’t significant. Rectangles and rhomboids eliminate undesirable drift d’ into the radiographic method, Figure 6b. Examples in Figure 7 clearly explain possibilities how the approach could be applied to deformed vertebrae to find the centre of the vertebral body width.

## Results

1. In total, we have carried out 30 measurements of all series (0 to 45 degrees), of which 10 have been evaluated by the author, 5 + 5 by two experts in the scoliosis field and 10 by students of the Faculty of Physical Education and Sport, Charles University in Prague. Students were educated in the new radiographic method.

All measurements were statistically evaluated. Standard deviation SD is for every group of evaluators in Table 1. Courses of arithmetic averages of all finding are recorded in Table 2.

**Table 1:** Overview of results of intra-individual and inter-individual precision determination, SD is a standard deviation

**Evaluation by etage**	**Author SD**	**Experts SD**	**Students SD**
Thoracic	1,33	1,86	2,73
L1-L4	1,27	2,21	2,99
L5	1,14	1,95	3,79
All	1,25	2,01	3,17

**Table 2:** Average values of all separate axial vertebral rotation (thoracic and lumbar columns together) which were evaluated (5580 pictures) by all individuals (author, 2 experts, 10 students)

**Average values of all axial vertebral rotation**	
**Defined**	**Evaluated**
0	1,3
3	3,0
6	6,1
9	8,8
12	12,4
15	15,4
18	19,0
21	21,6
24	24,5
27	27,6
30	30,3
33	33,1
36	36,0
39	38,4
42	41,6
45	44,2

Standard deviation SD = 2,42.

SD = 2.42 (5580 evaluations).

The table presents intra-individual finding of axial rotation separately by the author and two groups of inter-individual checks by experts and students. All arithmetic averages correlate very closely with the ideal values. Standard deviations SD of all groups show the relevancy and the legitimacy of the radiographic method.

2. In order to verify our method, we also used not only our own but also a published X-ray picture with defined rotational values [21]. We measured a picture of a thoracic vertebra rotated to 30°. Ten measurements carried out by the author (intra-individually) showed the value of 27.6°, SD = 0.6°. Inter-individual measurements gave the result of 27.9°, SD = 0.9°. The same picture was read by a Perdriolle torsion-meter with the result 34° (between 30° to 40°).

3. Some of our other evaluations of axial vertebral rotation by the new radiographic method were compared to the Perdriolle method, too. Several values of rotation (9, 18, 30 degrees) were chosen for thoracic and lumbar vertebrae which were measured using both methods. The comparison is presented in Table 3. Values in the table correlate appropriately. The results of both methods are comparable.

**Table 3:** Comparison of the new radiographic method to Perdriolle method in chosen defined rotation (9, 18 and 30 degrees)

**[Degrees]**	**Defined**	**Radiographic**	**Perdriolle**
**Th5**	9	8	9
	18	17	25
	30	33	45
**Th6**	9	8	9
	18	19	25
	30	31	40
**L1**	9	9	13
	18	18	24
	30	32	36
**L3**	9	5	10
	18	19	22
	30	30	30
**L5**	9	11	12
	18	19	20
	30	31	35

**Figure 8:**
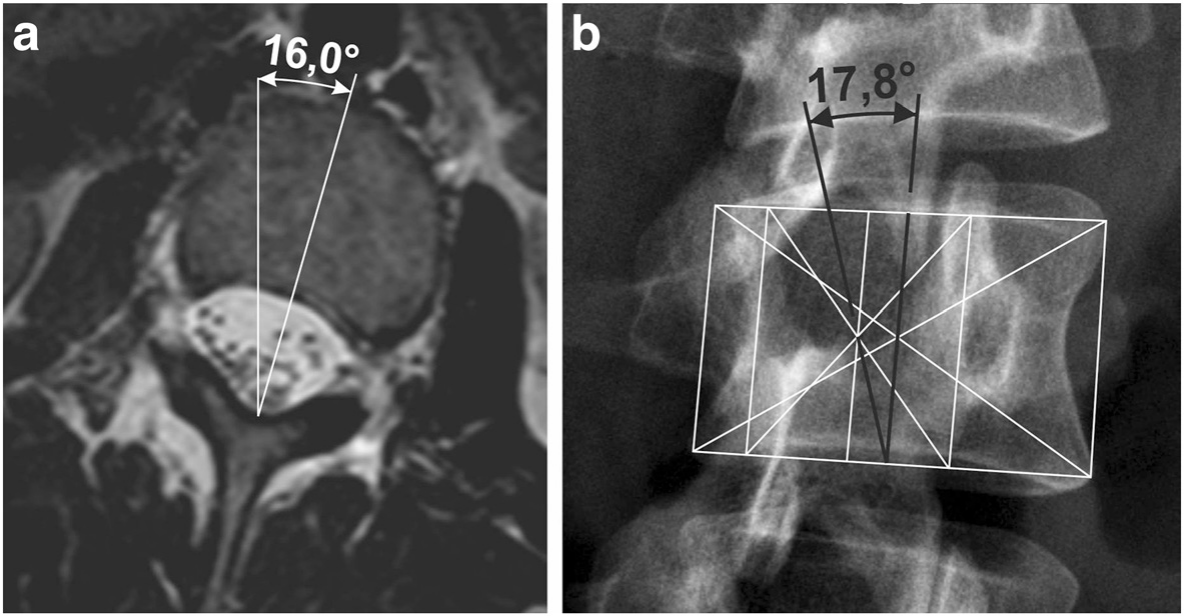
**Comparison of vertebral rotation measurement.** MRI picture in transversal plane **(a)**. Evaluation of axial vertebral rotation by the new radiographic method - table 3, case 2, vertebra L2 **(b)**.

4. Verification of axial vertebral rotation was performed with the assistance of MRI pictures of six patients (provided in supine position) and their comparison with the rotation measured by both the new radiographic method and the Perdriolle method at films of the same patient’s spine taken in supine position. The first one is a spine in segments TH12 to L5, the second one a lumbar spine in segments L1, L2, L4. Results of the three methods are presented in Table 4. All three methods showed very close degrees of rotation.Figure 8 shows an example of measurement. The degree of rotation measured by the new radiographic method is very close to the measurement of vertebra rotation at the transverse MRI picture.

**Table 4:** The comparison presents the new radiographic method to MRI and to Perdriolle method

	**Vertebra**	**Radiographic**	**MRI/CT**	**Perdriolle**
Case 1	Th12	30	29	30
	L1	34	32	33
	L2	34	32	33
	L3	30	28	28
	L4	26	26	20
	L5	12	16	15
Case 2	L1	14	13	17
	L2	18	16	22
	L4	8	8	15
Case 3	Th3	9	13	8
	Th6	7	7	8
	Th8*	16	9	25
Case 4	Th12	34	28	45
Case 5	Th5	4	9	0
Case 6	Th8*	11	7	20
	Th9*	14	9	22
	Th10*	10	8	15
	L2*	13	21	15
	L3	16	20	15
	L4	11	20	13

There are six cases with X-rays in supine position and MRI transverse pictures (supine position, too). *signs structural deformities, wedged vertebral body.

## Discussion

The new radiographic method for the evaluation of axial vertebral rotation in the antero-posterior views of conventional X-rays was compared to other methods which are used in clinical practice. The results presented demonstrate that our method is viable and sufficiently precise even in cases of scoliotic vertebrae – Figure 6a, b. The users of this new method are able to evaluate the axial rotation without any special aids.

The Nash-Moe method [8] defines the rotation very inaccurately, but it can be used for simple evaluation of vertebral rotation of the growing spine.

The Perdriolle and Raimondi methods provide more accurate values of axial vertebral rotation [12]. This accuracy is very useful for practice however the application of special devices for evaluation of vertebral rotation is necessary. Both methods use only life-l size vertebrae on AP projection of X-ray films. The Perdriolle method shows the rotation angles on lumbar spine very accurately but on the thoracic spine there is bigger difference to the real rotation. Geometrical shapes of thoracic and lumbar vertebrae are slightly different and only one torsion-meter is used for measurement of the spinal column. Separate series of both thoracic and lumbar spines show more accurate value for thoracic columns, see Table 3. The new radiographic method also uses readings of pedicle shadows and in a graphical way (without a torsion-meter) obtains similar values to the Perdriolle method.

A computer method according to Wei-Min Chi at al. [11] seems to be a very accurate method of evaluation of axial vertebral rotation from X-ray pictures in antero-posterior projection. The computer method is largely based on pedicle shadows again. However it is necessary to use special software and only digital pictures.

The newly presented radiographic method correlates very closely, as real angle values according to vertebral rotation devices as with MRI pictures, see Table 4. The evaluation of axial vertebral rotation with the assistance of MRI pictures of six scoliotic patients is the most convincing verification of our method.

Another advantage of the radiographic method is a possibility to evaluate the axial vertebral rotation not only from conventional X-ray films but also from their copies in a random size [21] and from digital pictures too. It’s universal.

The method has two slightly different ways of measurement procedure – for thoracic (and L5) and additionally for lumbar spines. The new radiographic method is affected by the same mistakes of morphologic variability [1] as the other previously mentioned methods which use pedicle shadows [2,3,6-11,22]. Shadows of vertebral bodies and their pedicles must be visible.The new radiographic method has some limitations. Pedicles become invisible on the concave side when the rotation exceeds 30°. It means that the method is applicable to values of rotation up to 30° approximately. In cases of markedly deformed scoliotic vertebrae we suppose less accurate values of axial rotation, too. In these cases it is necessary to substitute the deformed body contour with a rectangle or rhomboid to get both the centre of the vertebral body and the centre of pedicle, see Figure 6.

The radiographic method for the evaluation of axial vertebral rotation gives relevant and accurate results in the beginning of scoliosis during the growth of children when the contour of the vertebral bodies and pedicles are highly visible and the vertebrae are not so deformed. Accuracy of measurement is of course influenced by the experience of the evaluator. The method is very useful for the monitoring of axial vertebral rotation progression and the evaluation of basic rectification treatment (physiotherapy and bracing) or surgery.

## Conclusion

In the original paper the authors present their own new radiographic method that is sufficiently precise for clinical evaluation of the rotation of thoracic and the lumbar vertebrae in conventional X-rays.

The gold standard of the new radiographic method is in the evaluation of axial rotation of vertebrae to 30° approximately and the shape of vertebral bodies without severe structural deformities.

The main advantage of the presented radiographic method is the measurement of vertebral rotation from AP projection of conventional X-ray pictures only or from its printed copies with the use of simple readily available tools. The absolute size of X-ray pictures or their copies is not important for the accuracy of this method.

The new radiographic method seems to be suitable for the use in clinical practice because of simple measurement and sufficient accuracy.

## Nomenclature

***α*** … axial rotation of vertebra

***d*** … distance – hypotenuse of triangle with angle ***α***

***d’*** … drift of vertebral body centre of wedged vertebra

***x*** … radius of rotation in transversal plane

***z*** … height of vertebral body

* … multiplication

**~** … approximately equal to

***ǂ*** … parallelism of lines on figures

***LED*** … lower end-plate depth [20]

***SD*** … standard deviation (**σ**)

***SCD*** … spinal canal depth [20]

***tan*** … tangent

***VBHp*** … vertebral body height posterior [20]

## Competing interests

The authors declare that they have no competing interests.

## Authors’ contributions

All authors read and approved the final manuscript.
